# Fetal metabolic influences of neonatal anthropometry and adiposity

**DOI:** 10.1186/s12887-015-0499-0

**Published:** 2015-11-10

**Authors:** Jean M. Donnelly, Karen L. Lindsay, Jennifer M. Walsh, Mary Horan, Eleanor J Molloy, Fionnuala M. McAuliffe

**Affiliations:** UCD Obstetrics and Gynaecology, School of Medicine and Medical Science, University College Dublin, Dublin, Ireland; Department of Paediatrics, University of Dublin, Dublin, Ireland; Department of Neonatology, Our Lady’s Children’s Hospital Crumlin, Dublin, Ireland; National Maternity Hospital, Dublin, Ireland

**Keywords:** Adiposity, Anthropometry, Leptin, Maternal, Neonatal

## Abstract

**Background:**

Large for gestational age infants have an increased risk of obesity, cardiovascular and metabolic complications during life. Knowledge of the key predictive factors of neonatal adiposity is required to devise targeted antenatal interventions. Our objective was to determine the fetal metabolic factors that influence regional neonatal adiposity in a cohort of women with previous large for gestational age offspring.

**Methods:**

Data from the ROLO [Randomised COntrol Trial of LOw Glycaemic Index in Pregnancy] study were analysed in the ROLO Kids study. Neonatal anthropometric and skinfold measurements were compared with fetal leptin and C-peptide results from cord blood in 185 cases. Analyses were performed to examine the association between these metabolic factors and birthweight, anthropometry and markers of central and generalised adiposity.

**Results:**

Fetal leptin was found to correlate with birthweight, general adiposity and multiple anthropometric measurements. On multiple regression analysis, fetal leptin remained significantly associated with adiposity, independent of gender, maternal BMI, gestational age or study group assignment, while fetal C-peptide was no longer significant.

**Conclusion:**

Fetal leptin may be an important predictor of regional neonatal adiposity. Interventional studies are required to assess the impact of neonatal adiposity on the subsequent risk of childhood obesity and to determine whether interventions which reduce circulating leptin levels have a role to play in improving neonatal adiposity measures.

**Electronic supplementary material:**

The online version of this article (doi:10.1186/s12887-015-0499-0) contains supplementary material, which is available to authorized users.

## Background

Many factors, both maternal and environmental, are known to affect birthweight [1]. Infant size at birth is widely accepted to be an important determinant of later adult health [2], with neonates at both ends of the birthweight spectrum at risk of future health complications. Birthweight, and in particular the incidence of large for gestational age (LGA) infants, is increasing in most populations [3–5] which may be attributed to the increasing prevalence of maternal obesity and gestational diabetes mellitus [GDM] [4]. GDM incidence has estimated increases of 10 to 100 % for varying races and ethnicities over the last 20 years [6, 7]. Birthweight however is unlikely to be the best method of assessing nutritional status in the neonate and the resultant health impact. Previous research by Muthayya et al. [8] has shown that for the same birth weight different populations can different in their percentage of fat mass to lean mass, hence birthweight alone is a crude estimate of adiposity in children.

Not all LGA infants are born to diabetic mothers however. Women with normal glucose tolerance are also at risk of delivering larger weight babies at term [9]. Prevention of LGA in the euglycaemic population is therefore an area of increasing interest. We recently performed a RCT of low glycaemic index diet in pregnancy [10] (ROLO Study), and while it was not found to reduce the incidence of LGA infants in a group already at risk of fetal macrosomia, it did, however, have a significant positive effect on maternal glucose intolerance and maternal gestational weight gain.

Birth of a LGA or macrosomic infant presents a variety of obstetric and perinatal complications including increased risk of caesarean or instrumental delivery, transfer to a neonatal intensive care unit, shoulder dystocia [11], neonatal hypoglycaemia [12], and hip subluxation [13]. Furthermore, the developmental origins of adult disease hypothesis describes how large size at birth may predispose to early childhood obesity [14] and the metabolic syndrome and cardiovascular disease in later life [15, 16]. Thus, interventions to reduce the incidence of LGA and promote healthy birthweight are urgently required to improve pregnancy and future health outcomes. Birthweight alone however is a relatively crude assessment of neonatal adiposity. Greater knowledge of body composition and fat distribution in neonates may help to ascertain specific risk models that predict future childhood obesity and metabolic ill-health [17], which can subsequently be targeted in antenatal interventions.

While air displacement plethysmography is a validated form of adiposity measurement in neonates [18] this is often not practical or available. Anthropometric measurements including abdominal, chest and thigh circumferences and subscapular, biceps, triceps and thigh skinfold thicknesses, offer a simple alternative for assessing neonatal adiposity in the clinical setting [19, 20].

Leptin is an adipocyte secreted circulating polypeptide hormone expressed in the adipose tissue [21] that was first discovered in 1994. In adults it has a role to play in energy modulation of the body via a negative feedback mechanism between the centre of satiety in the hypothalamus and the adipose tissue [22]. In normal physiological conditions, it acts to promote energy expenditure and to inhibit feeding [23]. In utero the role of leptin is less clear. While the majority of leptin production occurs in the aforementioned adipocytes, leptin may also originate from other fetal tissues including the placenta [24, 25]. It is not clear however if placental-derived leptin has any role to play in fetal growth with some research suggesting that the majority of placental derived leptin may infact transfer to the maternal circulation [26]. A relationship between cord leptin and birthweight has previously been demonstrated by Lawlor et al. [27] and Clapp et al. [28]. As yet however the effect of leptin on individual areas of adiposity as determined by skinfolds has not been determined to our knowledge and its role as a growth factor remains inconclusive. As the expression of leptin is widespread in fetal tissue it suggests that it has an important role to play in fetal development [24].

C-peptide results from the transformation of proinsulin into insulin and was first discovered in 1967 with the discovery of the insulin synthesis pathway [29] and is an established effective marker of insulin secretion. Pedersen established the link between maternal glucose homeostasis and the fetal pancreatic response in 1952 [30]. It has been established that while glucose crosses the placenta, insulin cannot and therefore maternal hyperglycaemia causes a subsequent fetal hyperglycaemia and hyperinsulinaemia resulting in increased birthweight [31, 32]. Previous studies have also reported correlations between levels of cord C-peptide and birthweight [33–35].

To date, antenatal lifestyle interventions that target maternal nutrition and exercise in pregnancy have demonstrated limited effects on improving birthweight [36]. However, there is evidence for neuroendocrine fetal programming of birthweight through circulating maternal and fetal adipocytokines [37], which requires further investigation to ascertain the optimal interventions that may target these mechanisms. Previous studies have identified a positive association between fetal leptin and birthweight [38–40]. Consistent with the findings of Sewell et al. [41] Catelano et al. [42] determined that the offspring of obese mothers have an increased fat mass and percentage body fat compared with offspring of lean mothers and that increased fetal adiposity and fetal insulin resistance are closely associated hence the decision to study cord C-peptide along with cord leptin and its potential association with adiposity.

There is a paucity of literature investigating the link between fetal metabolic markers of obesity and more in-depth anthropometric measures of neonatal adiposity, which could provide valuable insight into the fetal origins of future obesity and metabolic disease. The only two studies we identified [43, 44] that looked at anthropometry in detail were in cohorts not at risk of macrosomia and subsequently early childhood obesity.

Therefore, the aim of this study was to determine the association between fetal metabolic factors and individual areas of neonatal adiposity, with a specific focus on fetal leptin and cord C-peptide in a cohort at high risk of macrosomia and early childhood obesity with a mean birthweight of greater than 4 kg.

## Methods

This is a follow on study of 185 infants born to women from the ROLO randomised controlled trial on whom cord blood C-peptide and leptin data were available as well as anthropometry at birth. Skinfold measurements were available for 147. The ROLO study was a randomised controlled trial of a low GI dietary intervention versus usual care among 800 non-diabetic, secundigravida women with a history of macrosomia, with the primary objective of reducing birthweight. Bloods including C-peptide and leptin were taken in early and late pregnancy and fetal sample from cord blood. Detailed methodology [45] and findings of the ROLO Study have been previously published [10]. The low GI dietary intervention in this study had no impact on birthweight or other neonatal outcomes, including various neonatal anthropometric measures except for thigh circumference [46]. The original ROLO trial had appropriate ethics approval from the National Maternity Hospital Ireland. This follow on trial had appropriate institutional ethics approval from Our Ladys’ Children’s Hospital Crumlin and the National Maternity Hospital Ireland and written informed consent from all patients involved or their guardian in the case of the offspring, and was performed in accordance with the ethical standards laid down in the 1964 Declaration of Helsinki and its later amendments.

### Data collection

At their first antenatal consultation (13.0 ± 2.3 weeks), all participants had their weight and height recorded and their body mass index (BMI) calculated. Whether or not the mothers had achieved third level education was recorded as a marker of socioeconomic status. Father’s height was recorded if fathers were present at the consultation or if mothers were sure of their partner’s height when asked. Father’s weight was recorded if they attended an antenatal consultation. Cord bloods were collected at delivery and neonatal anthropometric measurements were taken during the first 72 h of life including; birthweight, length, occipital frontal head circumference (OFC), chest, abdominal, thigh and mid upper arm circumferences. Each measurement was taken by a trained operator in triplicate and the average result recorded. To ensure standardisation, one in three neonates had measurements repeated by a second trained observer and the average value was compared to that of the first operator [20]. Measurements were repeated for a third time (*n* = 3) if any large discrepancy (>1 cm) was noted between the observers. Birthweight was measured on a Seca calibrated scales. A Seca lasso tape [47] was used for all circumferences and crown heel length was also recorded on a Seca measuring board [20]. A subgroup (among total ROLO population) also had skinfold measurements obtained by a trained observer using Holtain callipers, which included biceps, triceps, subscapular and thigh skinfold thicknesses [48]. Measurements were recorded following the ROLO Kids Standard Operating Procedure which was based on the National Health and Nutrition Examination Survey procedure [49].

Subscapular-to-triceps skinfold ratio (SS/TR) [50] and waist-to-height ratio [51] were calculated and used as markers of central adiposity while the sum of all skinfolds and subscapular plus triceps skinfolds [SS + TR] were used as markers of general adiposity [50].

### Laboratory methods

Multianalyte profiling was performed on the Luminex Magpix system (Luminex Corporation, Austin, USA.). Fetal insulin resistance was assessed via cord blood C-peptide estimation. Plasma concentrations of leptin and C-peptide were determined by the Human Endocrine Panel.

### Statistical analysis

All statistical analyses were performed using SPSS version 20.0 (SPSS Inc., Chicago, IL). Data were assessed for normality by visual inspection of histograms. Descriptive statistics were employed to describe baseline maternal and neonatal characteristics, cord blood C-peptide and leptin concentrations and neonatal anthropometry. Differences in each of these baseline and anthropometric variables between the original ROLO Study intervention and control groups were assessed using the independent samples *t*-test and the chi-squared test for continuous and categorical variables, respectively. Correlations between cord blood C-peptide and leptin and each of the neonatal anthropometric measures were analysed. Significantly correlated variables (*p* < 0.05) including maternal leptin measured in early and late pregnancy (which may have influenced cord leptin levels) were subsequently analysed by simple linear regression, with each anthropometric measure as the dependent variable, and then imputed into multiple linear regression models using a combination of forced entry and backwards stepwise procedures. Factors known to influence neonatal size [52, 53], i.e. total gestational age, infant gender, maternal BMI and maternal educational level (as a marker of socioeconomic status) were included in the models as forced entry variables. As this was a secondary analysis of a randomized trial, the original group assignment (dietary intervention vs. no intervention) from the ROLO study was also included as a forced entry variable. The final model was used as the best predictor of the change in the dependant variable.

## Results

The mean maternal BMI was 27 kg/m^2^, and the mean birthweight, was above 4 kg indicating that this is a macrosomic cohort of neonates (Table 1). Total gestational age differed significantly between the ROLO study groups, such that infants born to women in the intervention group were more gestationally mature compared to infants of control group women (Table 1). The neonatal anthropometric measures did not differ between the ROLO study intervention and control groups (Additional file 1: Table S1).

**Table 1:** Baseline characteristics among the total sample and by intervention group of the original ROLO study

	Total	Intervention	Control	*P*-value
	(*n* = 185)	(*n* = 89)	(*n* = 96)	
	Mean (SD^a^)			
Maternal age (years)	32.6 (4.2)	33.0 (3.8)	32.4 (4.3)	0.415
Maternal early pregnancy BMI^a^ (kg/m^2^)	26.9 (4.7)	27.4 (4.9)	26.6 (4.5)	0.267
Total gestational age (days)	282.9 (7.2)	284.1 (7.0)	281.7 (7.3)	0.022
Neonatal birthweight (kg)	4.08 (0.48)	4.11 (0.51)	4.05 (0.45)	0.414
	Median (IQR^a^)			
Cord C-peptide (ng/ml)	567.13 (4106.2)	619.66 (4106.2)	562.67 (3044)	0.0.991
Cord leptin (ng/ml)	27.40 (28.6)	29.8 (30.68)	26.66 (28.60)	0.0.978
	N (%)			
Mother smoked in pregnancy	5 (2.7)	3 (3.4)	2 (2.2)	0.596
Mother achieved third level education education	100 (54.1)	42 (47.2)	57 (59.4)	0.104
Male baby	85 (45.9)	44 (49.4)	41 (42.7)	0.322

^a^BMI, body mass index; IQR, interquartile range; SD, standard deviation. *P*-values calculated by the independent samples *t*-test for normally distributed continuous variables, Mann–Whitney *U* test for non-normal continuous variables (cord C-peptide and leptin) and the chi-squared test for categorical variables

Cord blood C-peptide significantly positively correlated with all skinfold thickness measures, sum of all skinfolds and sum of subscapular and triceps skinfold (see Additional file 2: Table S2). Cord blood leptin was significantly positively correlated with all anthropometric measures except head circumference and subscapular-triceps skinfold ratio. There was no significant difference in the degree of association between cord leptin and each circumference or skinfolds using simple linear regression (Additional file 2: Table S2).

Multiple linear regression analysis determined the most significant predictive model for each outcome variable [Table 2]. Fetal leptin was the most significant determinant of each of the anthropometric markers including abdominal, thigh, chest and arm circumferences. It was also the most significant determinant in the final model for waist-height ratio, a marker of central adiposity, overall neonatal adiposity as determined by SS + TR and sum of skinfolds, independent of maternal BMI, neonatal gender, total gestational age, maternal education or study group assignment. There was no difference noted in the degree of association between the individual circumferences and skinfolds and fetal leptin. All were significantly associated with fetal leptin with (*p* < 0.05). Earlier associations of C-peptide with neonatal anthropometry became non-significant in the multiple regression models perhaps due to the effect being attenuated by the cord leptin.

**Table 2:** Multiple linear regression of the association between neonatal anthropometry and cord blood C-peptide and leptin

	B	S.E.B.	*P*-value	R^2^	F	*P*-value
Birthweight (Kg)						
Fetal Leptin	7.611	3.036	0.017	0.422	5.688	<0.001
Father’s Weight	13.381	5.607	0.022			
Abdominal circumference (cm)						
Fetal Leptin	0.038	0.010	<0.001	0.112	3.137	0.005
Leptin 1^st^ Trimester	0.057	0.025	0.022			
Thigh circumference (cm)						
Fetal Leptin	0.017	0.009	0.046	0.073	2.072	0.066
Chest circumference (cm)						
Fetal Leptin	0.031	0.011	0.006	0.059	2.556	0.024
Mid-upper arm circumference (cm) (cm)						
Fetal Leptin	0.018	0.005	<0.001	0.105	3.888	< 0.001
Waist-height ratio						
Fetal Leptin	0.001	0.000	0.015	0.135	1.83	0.132
Subscapular skinfold thickness (mm)						
Fetal Leptin	0.029	0.007	<0.001	0.312	5.142	<0.001
Early Pregnancy Leptin	−0.054	0.017	0.002			
Triceps skinfold thickness (mm)						
Fetal Leptin	0.025	0.010	0.019	0.177	2.755	0.017
Late Pregnancy Leptin	−0.037	0.016	0.024			
Biceps skinfold thickness (mm)						
Fetal Leptin	0.027	0.006	<0.001	0.137	3.637	0.003
Thigh skinfold thickness (mm)						
Fetal Leptin	0.035	0.007	<0.001	0.173	5.414	<0.001
Sum of all skinfolds (mm)						
Fetal Leptin	0.130	0.022	<0.001	0.296	6.250	<0.001
SS + TR skinfold thickness (mm)						
Fetal Leptin	0.055	0.014	<0.001	0.253	4.195	<0.001
Late Pregnancy Leptin	−0.054	0.022	0.018			

SS = Subscapular, TR = Triceps, SS/TR = Central Adiposity SS + TR = General Adiposity, SF = skinfolds. All Multiple Regression analysis included Maternal BMI, Group, Gender, Total Gestation and Maternal Education Level of achievement as Enter variables. Anthropometry was the dependant variable. Only independent variables with a significant effect [*p* < 0.005] on the dependant variable as determined via simple linear regression were included in the multiple linear regression analysis. S.E.B. is the standard error of the computed value of b

While no universal agreement exists regarding the use of multiple testing corrections, the multiple regression results for birthweight, mid upper arm circumference, subscapular skinfold, thigh skinfold, sum of skinfolds and subscapular plus triceps skinfold thickness would all have survived a Bonferroni correction for the 23 [original] predictors examined (*p* < 0.0022).

## Conclusion and discussion

We have comprehensively examined regional neonatal anthropometry and its relationship to fetal leptin and fetal C-peptide in a European cohort at risk of macrosomia and subsequent childhood obesity. As the average birthweight is increasing internationally it is important to examine the influence of fetal metabolic parameters in this cohort and the individualised effect of these markers on regional areas of adiposity.

While previous studies have shown a connection between cord leptin and birthweight [27, 28] few have looked at individualised anthropometric markers and hence a true connection with cord leptin and regional areas of neonatal adiposity. Two previous studies have ascertained a link between fetal leptin and neonatal anthropometry, but cord C-peptide was not included in the analysis. The first involved a cohort with an average birthweight of 3.77Kg [43] conducted in China in 2004 and the second [44] a cohort with an average birthweight of 2.95Kg in Lithuania. We believe our study is the largest study to examine the correlation between cord leptin and cord C-peptide on individual markers of neonatal adiposity in a cohort at risk of early childhood obesity.

These data confirm the association between cord leptin and individual markers of neonatal adiposity. While early pregnancy maternal leptin was associated with neonatal abdominal circumferences and neonatal subscapular skinfold thicknesses, and late pregnancy leptin levels with triceps skinfolds and the SS + TR marker, it is fetal leptin which is most significantly associated with birthweight, individual markers of regional adiposity and also markers of central and general adiposity. On multiple regression analysis, fetal leptin remained positively associated with neonatal anthropometry, independent of BMI, neonatal gender total gestational age or study group assignment. There was no significant difference in the degree of association between fetal leptin and the various individual markers of adiposity. This suggests that the effect of leptin on adiposity is generalised and not limited to central or peripheral adiposity as determined by the various markers measured in our study. Fetal C-peptide was no longer significantly associated with any adiposity measures however fetal C-peptide was strongly correlated with cord leptin (correlation coefficient 0.415, *p* < 0.001) which may explain why c-peptide was not determined to be an independent correlate of the anthropometry .

What remains unclear however is, if fetal leptin is higher in those with larger skinfolds and circumferences simply as it is secreted by the fetal adipose tissue or if it has a roll to play as a growth factor. Previous research has shown that neonates born LGA do indeed have higher levels of cord blood leptin than those born small for gestational age [54] and that fetal leptin is also a predictor of fat mass at birth [55] and at 3 years of age [35]. Hassink et al. [56] showed that serum leptin concentrations in newborns were increased more than three-fold compared with children in the early stages of puberty when controlled for adiposity, therefore suggesting that leptin concentrations in the newborn were not explained by adiposity alone. Prior research has also shown that leptin receptors are widely located in the developing fetus suggesting that leptin is involved in fetal growth e.g. Javaid et al. [57]. At present while no specific studies have proven that fetal leptin is a major growth factor in fetal development, the majority of these studies, as with ours, have been conducted using gross markers of adiposity e.g. birth weight. It may be then that the influence of leptin as a growth factor is at the cellular level. A large review of the role of leptin as a nutritional signal by Forhead et al. [24] concluded that while the roll of leptin as a growth factor remains unclear, the widespread expression of leptin suggests that leptin has physiological significance in fetal life. This is consistent with our findings that fetal leptin is associated with birthweight and adiposity as determined by individual anthropometry in the neonatal period.

This was a well characterised cohort from the ROLO study, which was a randomised controlled study examining neonates born to non-diabetic women with a history of a previous macrosomic child. As the ROLO Study did not find any difference in the birthweight [10] or adiposity measures except the aforementioned thigh circumference [46] of the neonates between intervention and control groups, this has facilitated follow-up analysis of the offspring as a cohort of healthy babies born to non-diabetic healthy mothers. The current study is strengthened by the use of multiple regression analysis and inclusion of a variety of neonatal anthropometric measurements to assess both generalised and central adiposity.

Our study has some limitations worthy of consideration. As previously mentioned, participants of the ROLO study were all secundigravida and non-diabetic. Although women who had previously been diagnosed with gestational diabetes were excluded, as genetics are known to play a role in the determination of birthweight, the cohort may have had a selection bias in choosing women who were more likely to have a larger birthweight baby.

The intervention group in the ROLO study were instructed to change to a low GI diet. Although this intervention was shown not to affect birthweight or leptin concentrations, the reduced gestational weight gain among the intervention group [58] and conscious diet alternation of the unblinded control group may have moderated the fetal leptin results. Additionally while the equations of subscapular plus triceps skinfolds and subscapular/triceps ratio are widely accepted to correlate closely with DXA measurements of fat mass [59, 60] they have not specifically been validated in our age group.

Our findings have contributed to a growing body of evidence that the fetal metabolic milieu plays a vital role in the determination of neonatal adiposity at birth in non-diabetic pregnancies and that fetal leptin may be a key metabolic factor to target in these pathways. Interventional studies are required to assess the impact of neonatal adiposity on the subsequent risk of childhood obesity and to determine whether interventions which reduce circulating leptin levels have a role to play in improving neonatal adiposity measures.

## Additional files

Additional file 1: Table S1. Baseline anthropometric measurements among the total sample and by intervention group of the original ROLO study (DOC 39 kb) Additional file 2: Table S2. Correlation of fetal C-peptide and leptin from cord blood with neonatal anthropometric measures (DOC 53 kb)

## Abbreviations

BMI: Body mass index
GDM: Gestational diabetes mellitus
GI: Glycaemic index
LGA: Large for gestation age
OFC: Occipital frontal head circumference
ROLO: Randomised COntrol Trial of LOw Glycaemic Index in Pregnancy
SGA: Small for gestational age
SS: Subscapular
TR: Triceps
