# Supplementary material for: Fetal metabolic influences of neonatal anthropometry and adiposity
Source: BMC Pediatr. 2015 Nov 10;15:175. doi: 10.1186/s12887-015-0499-0 (PMC4641416; doi:10.1186/s12887-015-0499-0)
Supplement: Additional file 1: Table S1. — Baseline anthropometric measurements among the total sample and by intervention group of the original ROLO study (DOC 39 kb) [file 12887_2015_499_MOESM1_ESM.doc]

**Additional file 1: Table S1: Baseline anthropometric measurements among the total sample and by intervention group of the original ROLO study**

|  | **Total**  **(n=185)** | **Intervention**  **(n=88)** | **Control**  **(n=93)** | **P-Value** |
| --- | --- | --- | --- | --- |
|  | **Mean (SD**^*^**)** | | |  |
| Head circumference (cm) | 35.80 (1.22) | 35.95 (1.27) | 35.66 (1.16) | 0.124 |
| Abdominal circumference (cm) | 33.48 (2.21) | 33.42 (2.42) | 33.53 (2.01) | 0.733 |
| Thigh circumference (cm) | 16.19 (1.39) | 16.14 (1.55) | 16.23 (1.22) | 0.653 |
| Chest circumference (cm) | 35.62 (2.83) | 35.35 (2.48) | 35.86 (3.11) | 0.216 |
| Mid-upper arm circumference (cm) | 12.43 (1.26) | 12.43 (1.52) | 12.42 (0.97) | 0.933 |
| Waist-height ratio | 0.635 (0.05) | 0.63 (0.04) | 0.64 (0.05) | 0.121 |
| Subscapular skinfold thickness (mm) | 6.93 (1.57) | 6.92 (1.60) | 6.94 (1.55) | 0.928 |
| Triceps skinfold thickness (mm) | 7.02 (1.52) | 6.99 (1.58) | 7.05 (1.48) | 0.838 |
| Biceps skinfold thickness (mm) | 6.78 (1.50) | 6.70 (1.50) | 6.85 (1.50) | 0.565 |
| Thigh skinfold thickness (mm) | 8.07 (1.85) | 8.16 (2.07) | 7.99 (1.64) | 0.582 |
| Sum of all skinfolds (mm) | 28.80 (5.38) | 28.78 (5.77) | 28.82 (5.05) | 0.958 |
| SS+TR^*^ skinfold thickness (mm) | 13.95 (2.73) | 13.91 (2.81) | 13.98 (2.67) | 0.868 |
| SS/TR^*^ skinfold ratio | 1.00 (0.20) | 1.01 (0.199) | 1.00 (0.19) | 0.854 |
| *BMI, body mass index; IQR, interquartile range; SD, standard deviation. P-values calculated by the independent samples t-test for normally distributed continuous variables, Mann-Whitney U test for non-normal continuous variables (cord C-peptide and leptin) and the chi-squared test for categorical variables. | | | | |
