# Supplementary material for: Fetal metabolic influences of neonatal anthropometry and adiposity
Source: BMC Pediatr. 2015 Nov 10;15:175. doi: 10.1186/s12887-015-0499-0 (PMC4641416; doi:10.1186/s12887-015-0499-0)
Supplement: Additional file 2: Table S2. — Correlation of fetal C-peptide and leptin from cord blood with neonatal anthropometric measures (DOC 53 kb) [file 12887_2015_499_MOESM2_ESM.doc]

**Additional file 2: Table S**2: Correlation of fetal C-peptide and leptin from cord blood with neonatal anthropometric measures

|  |  | **Cord blood C-peptide (ng/ml)** | **Cord blood Leptin (ng/ml)** |
| --- | --- | --- | --- |
| Birthweight (Kg) | Correlation coefficient | 0.113 | 0.346 |
|  | P-value | 0.129 | *<0.001* |
| Head circumference (cm) | Correlation coefficient | 0.071 | 0.106 |
|  | P-value | 0.343 | 0.173 |
| Abdominal circumference (cm) | Correlation coefficient | 0.140 | 0.297 |
|  | P-value | 0.058 | *<0.001* |
| Thigh circumference (cm) | Correlation coefficient | 0.133 | 0.326 |
|  | P-value | 0.070 | *<0.001* |
| Chest circumference (cm) | Correlation coefficient | 0.072 | 0.227 |
|  | P-value | 0.328 | *0.003* |
| Mid-upper arm circumference, straight (cm) | Correlation coefficient | 0.126 | 0.290 |
|  | P-value | 0.087 | *<0.001* |
| Mid-upper arm circumference, flexed (cm) | Correlation coefficient | 0.110 | 0.275 |
|  | P-value | 0.137 | *<0.001* |
| Waist-height ratio | Correlation coefficient | 0.081 | 0.309 |
|  | P-value | 0.314 | *<0.001* |
| Subscapular skinfold thickness (mm) | Correlation coefficient | 0.206 | 0.485 |
|  | P-value | *0.012* | *<0.001* |
| Triceps skinfold thickness (mm) | Correlation coefficient | 0.202 | 0.387 |
|  | P-value | *0.014* | *<0.001* |
| Biceps skinfold thickness (mm) | Correlation coefficient | 0.282 | 0.401 |
|  | P-value | *<0.001* | *<0.001* |
| Thigh skinfold thickness (mm) | Correlation coefficient | 0.225 | 0.455 |
|  | P-value | *0.006* | *<0.001* |
| Sum of all skinfolds (mm) | Correlation coefficient | 0.273 | 0.519 |
|  | P-value | *0.001* | *<0.001* |
| SS+TR skinfold thickness (mm) | Correlation coefficient | 0.232 | 0.495 |
|  | P-value | *0.005* | *<0.001* |
| SS/TR skinfold ratio | Correlation coefficient | 0.006 | 0.112 |
|  | P-value | 0.947 | 0.177 |
| SS+TR, subscapular plus triceps skinfold; SS/TR, subscapular-to-triceps skinfold ratio. Circumference measurements available for all neonates in study sample, skinfold measurements available for N=147. Pearson and Spearman Rho correlation coefficients used for normally and non-normally distributed variables, respectively. Variables are significantly correlated where p<0.05 and are shown in italics. | | | |
